# A rare case of hepatic subcapsular biloma after laparoscopic cholecystectomy and subsequent endoscopic retrograde cholangiopancreatography

**DOI:** 10.22088/cjim.9.2.198

**Published:** 2018

**Authors:** Alimohammad Jafari

**Affiliations:** 1 Department Of Internal Medicine, North Khorasan University of Medical Sciences, Bojnurd, Iran.

Bile leak after open or laparoscopic cholecystectomy is usually a result of minor biliary injury, although it can sometimes reveal a major duct injury. It is estimated that biloma originates from the cystic duct in more than 50% of the cases (1). Hepatic subcapsular biloma is a rare complication after cholecystectomy or ERCP. There are only some cases reported in the literature of this complication after laparoscopic cholecystectomy. Post ERCP biloma is an unusual complication which was reported in some literature (2). We describe a rare case of a 30-year-old woman, presented 4 weeks after laparoscopic cholecystectomy and 2 weeks after ERCP with symptom of a right upper quadrant pain, a result of subcapsular biloma diagnosed by a computed tomography (CT) and successfully treated by percutaneous drainage. 

## Case presentation

A 30-year-old previously healthy Iranian woman presented to the emergency department 4 weeks after laparoscopic cholecystectomy for acute cholecystitis and 2 weeks after ERCP with a 2 days history of worsening right upper quadrant pain.

After surgery she had mild right upper quadrant pain. Two weeks after laparoscopic surgery, the patient was admitted to the hospital due to a worsening pain in her right upper quadrant and according to dilated CBD in abdominal sonography, ERCP was performed with the probability of retained CBD stone in which sphincterotomy was done but only some sludge was retrieved from CBD. Two weeks after ERCP, she presented to the hospital and was admitted due to a worsening RUQ pain. She had low grade fever, mild right upper quadrant pain, no vomit and no jaundice. She had no prior history of abdominal surgery or trauma except laparoscopic cholecystectomy. 

**Figure 1:**
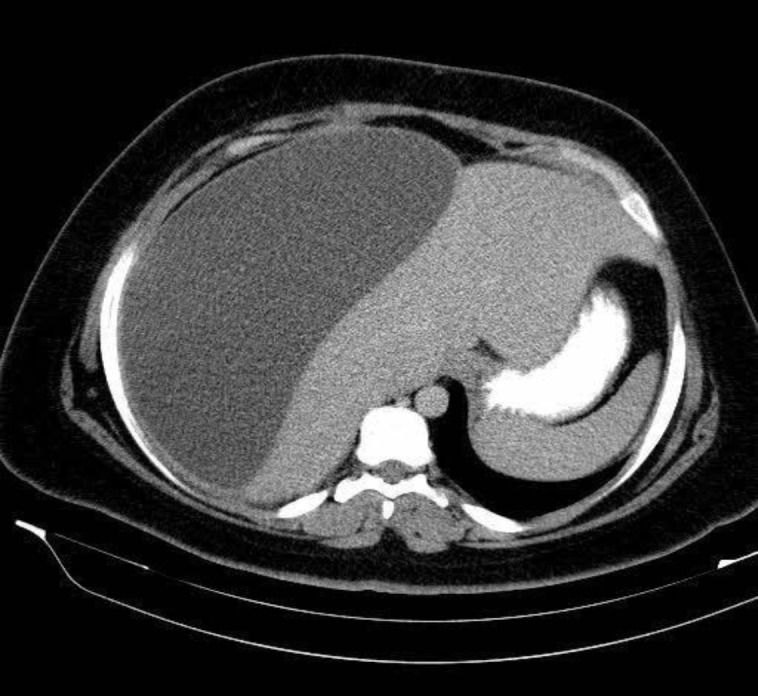
Abdominal CT (axial) demonstrated a large subcapsular biloma in the right lobe of the liver. Notable compression of underlying parenchyma is evident.

The physical examination revealed a conscious woman who had a 37.5 degree C temperature a pulse rate of 80 beats per minute (bpm), and a blood pressure of 120/70 mm Hg. The abdominal examination revealed a mild tenderness in the right upper quadrant. Laboratory data revealed an Hb of 10.6 g/dl, white blood cells of 9,900/mm³ (70% neutrophils), a BUN (blood urea nitrogen) of 10 g/dL, and a creatinine level of 0.8 mg/dL. Liver enzymes showed a total bilirubin of 1.7 mg/dL with a direct component of 0.4 mg/dL; AST was 20 U/L with normal range of <31 U/L in female and ALT was 14 U/L with normal range of <32 U/L for female. Transabdominal Ultrasonography (US) revealed a large 24*22 mm subcapsular fluid-filled collection in the right liver lobe. There was no free intra-abdominal fluid. Abdominal computed tomography (CT) confirmed results of US and concluded a large hepatic subcapsular fluid collection measuring (25 cm × 23 cm × 10 cm) most likely it was a subcapsular biloma (figures 1). 

Based on the imaging findings, we made a decision to perform a percutaneous US-guided fluid drainage which was done and drained bile appearance fluid was sent to the laboratory for chemical analysis. Characteristics of drained fluid were; WBC 0-1/mm^3^, bacteria not seen, culture negative, total bilirubin 10.4 mg/dl and direct bilirubin 2.2 mg/dl.

Therapeutic percutaneous puncture was done which removed 3500 ml of bile. After draining, catheter was placed into biloma site. ERCP was done and a 10 French 10 cm plastic stent was placed in CBD to control bile leakage. The catheter was drained less than 100 ml of bile over 3 days. Therefore, it was removed 2 days later when the output was ceased. A follow up US was done showing a near-complete resolution. Three weeks later we removed the CBD plastic stent. 

## Discussion

The term biloma was introduced in 1979 by Gould and Pater to describe a loculated collection outside the biliary tree (3, 4, 5). Kuligowska et al. extended the term biloma to include intrahepatic as well as extrahepatic collections of bile (6). Bilomas mainly result from iatrogenic, traumatic, or spontaneous rupture of the biliary ducts (7). Although bile leakage into the peritoneal cavity is a known complication of open and laparoscopic cholecystectomy [8], the hepatic subcapsular biloma is a rare complication after laparoscopic cholecystectomy and ERCP. Some authors attribute this complication to a small perforated radical biliary because of the backpressure associated with the high-pressure irrigation used during choledochoscopy (9). 

High pressure in the proximal biliary ducts, caused by injection of contrast material, is the reported cause of a hepatic subcapsular biloma after ERCP (2). Some authors attribute this complication in patients, with blunt liver truma (10) though we think that the possible etiology for the hepatic subcapsular biloma in our patient is an injury to small proximal biliary ducts because of high pressure, caused by injection of contrast material during ERCP or trauma to small biliary ducts, caused by the tip of the guide wire. Although rupture of small ducts during laparoscopic cholecystectomy was possible etiology for this complication in our patient. Why so, the patient had right upper quadrant pain and discomfort after surgery and although the CBD was dilated in imaging after surgery and presence of the retained stone was probable, nevertheless there was no stone extracted in first ERCP and it was possible that the pain was due to early phase of subcapsular bile accumulation and biloma establishment. The right upper quadrant abdominal pain is the most common symptom in the patient with subcapsular biloma, associated sometimes with nausea and vomiting (9). Ultrasound is sensitive for diagnosing bilomas, but the diagnosis of this complication is ideally facilitated by the use of Computed tomography and documented by fluid drainage and analysis (11). 

Imaging of the biliary tree should be performed early to determine the location and extent of bile leaks [9]. Hepatic subcapsular biloma can be drained percutaneously. The drainage catheter was removed when the output is minimal (9-13). Our patient was managed with US guided percutaneous drainage with a good response. In conclusion, a subcapsular biloma is a rare complication of laparoscopic cholecystectomy and ERCP. Early diagnosis and appropriate percutaneous drainage are the keys to manage this rare complication.
